# Identifying Dynamic Functional Connectivity Changes in Dementia with Lewy Bodies Based on Product Hidden Markov Models

**DOI:** 10.3389/fncom.2016.00060

**Published:** 2016-06-23

**Authors:** Marion Sourty, Laurent Thoraval, Daniel Roquet, Jean-Paul Armspach, Jack Foucher, Frédéric Blanc

**Affiliations:** ^1^Centre National de la Recherche Scientifique, FMTS, University of Strasbourg, ICube-UMR 7357Strasbourg, France; ^2^CEMNIS (Noninvasive Neuromodulation Center), University Hospital of StrasbourgStrasbourg, France; ^3^CMRR (Memory Resources and Research Center), University Hospital of Strasbourg, Geriatrics and Neurology ServicesStrasbourg, France

**Keywords:** dynamic functional connectivity, dynamic Bayesian networks, resting-state fMRI, product HMM, dementia with Lewy bodies

## Abstract

Exploring time-varying connectivity networks in neurodegenerative disorders is a recent field of research in functional MRI. Dementia with Lewy bodies (DLB) represents 20% of the neurodegenerative forms of dementia. Fluctuations of cognition and vigilance are the key symptoms of DLB. To date, no dynamic functional connectivity (DFC) investigations of this disorder have been performed. In this paper, we refer to the concept of connectivity state as a piecewise stationary configuration of functional connectivity between brain networks. From this concept, we propose a new method for group-level as well as for subject-level studies to compare and characterize connectivity state changes between a set of resting-state networks (RSNs). Dynamic Bayesian networks, statistical and graph theory-based models, enable one to learn dependencies between interacting state-based processes. Product hidden Markov models (PHMM), an instance of dynamic Bayesian networks, are introduced here to capture both statistical and temporal aspects of DFC of a set of RSNs. This analysis was based on sliding-window cross-correlations between seven RSNs extracted from a group independent component analysis performed on 20 healthy elderly subjects and 16 patients with DLB. Statistical models of DFC differed in patients compared to healthy subjects for the occipito-parieto-frontal network, the medial occipital network and the right fronto-parietal network. In addition, pairwise comparisons of DFC of RSNs revealed a decrease of dependency between these two visual networks (occipito-parieto-frontal and medial occipital networks) and the right fronto-parietal control network. The analysis of DFC state changes thus pointed out networks related to the cognitive functions that are known to be impaired in DLB: visual processing as well as attentional and executive functions. Besides this context, product HMM applied to RSNs cross-correlations offers a promising new approach to investigate structural and temporal aspects of brain DFC.

## 1. Introduction

Brain functional connectivity studies in resting-state functional MRI provide functional networks that correspond to spontaneously co-activated cerebral regions, also named resting-state networks (RSNs) when shared by a population. So far, interactions between brain areas have been described under the assumption of temporal stationarity of functional connectivity, producing a static description of those interactions. Recently, dynamic functional connectivity (DFC) investigations demonstrated that cerebral regions do not interact in a static way, but rather in a dynamic process that could change over time (Hutchison et al., 2013; Calhoun et al., 2014). These variations have been particularly highlighted by Chang and Glover (2010) with a time-frequency analysis that allows to investigate dynamic proofs in functional connectivity signal at different time scales. To go further in this new growing field of research, the studies are nowadays directed toward the concept of neural connectivity state, along with the possibility of reproducible patterns of DFC. Several methods have been developed to assess interactions between cerebral regions or networks mainly based on a sliding windows approach used with correlation (Leonardi et al., 2013; Allen et al., 2014) and spatial (Kiviniemi et al., 2011) or temporal (Smith et al., 2012) independent component analysis. But there is no consensus on the definition of “connectivity state,” and tools to measure and quantify this dynamics are still to be developed.

When considering the dialectic used in DFC analysis (“dynamic,” “brain state,” “graph,” “network,” etc.), it seems natural to think about methodological tools such as dynamic Bayesian networks (DBN), or instances of DBN such as hidden Markov models (HMM), or product HMM (PHMM), to capture temporal dependencies between RSN. Markov models (not “hidden” Markov models) have already been used in DFC analysis, but not as a primary tool, to point out different connectivity states from sliding-windows correlations on time-series of defined brain regions (Allen et al., 2014; Ma et al., 2014). To date, very few studies have used dynamic Bayesian networks, HMM, or product HMM to investigate DFC in resting-state fMRI (Eavani et al., 2013; Ou et al., 2013). For an exploratory purpose, Eavani et al. (2013) proposed a method to highlight, at subject level, the variability of functional connectivity. Applied on clustering covariance matrices from defined regions of interest, the HMM approach they proposed was able to decode DFC into temporal sequences of hidden states, each state associated with a distinct connectivity pattern. The authors introduced their algorithm with two to ten connectivity states allowing them to distinguish different brain patterns. Ou et al. (2013) used HMM on a large scale functional connectivity matrix to distinguish children with an attention deficit hyperactivity disorder from control children. With multiple initialization parameters, their method led to 15 to 25 hidden connectivity states that well described the two studied groups. In order to be computational, the cited methods introduced data reduction techniques to cope with the high dimension of fMRI data. Then, the number of states is usually limited either by the number of cerebral regions to include in the connectivity study, or by a clustering of functional connectivity regions performed prior to the Markov model analysis (Eavani et al., 2013; Allen et al., 2014; Ma et al., 2014).

The number of cerebral regions or RSNs to observe and thus the number of possible interactions are very numerous in DFC studies. This remark should be especially taken into consideration as fMRI studies provide a limited number of observations, making the robustness more difficult to ensure. To solve this problem, the methods mentioned above rely on a data reduction step. Connectivity states are defined by clustering according to what was observed during a first step of DFC analysis. This strategy leads to a small number of states and allows one to focus exclusively on the observed data. The states, defined in a group of subjects, represent spatial patterns of connectivity between RSNs that appear in a reproducible manner in time and/or across subjects. The dynamics between these states is thereafter modeled by a Markov chain. This procedure is assumed to sufficiently take into account, in the reduction and in the Markov modeling, spatial and temporal interactions between RSNs. However, this approach, although closer to the data, does not allow one to observe the states that are more ephemeral or less representative of the DFC of a group of subject. An alternative approach consists in defining the connectivity states, directly from the states in each RSN, without knowledge of which interactions will appear in the observed sequence. In this way, the complexity of these RSN interactions is transferred into a multi-dimensional modeling as proposed by product HMM. These states represent all the possibilities of interactions between the RSNs and are defined as the Cartesian product of the states sub-spaces in each process. Thereafter, the DFC is modeled by the product HMM. This large state space allowed by this multi-dimensional approach enables a better consideration of dynamic aspects without requiring a reduction of upstream data.

Within the product HMM framework, this paper compares brain DFC between patients with dementia with Lewy bodies (DLB) and healthy elderly controls. DLB is the second most prevalent form of neurodegenerative dementia after Alzheimer's disease, affecting from 16 to 20% of patients with dementia (Aarsland et al., 2008). The main clinical criteria of DLB are cognitive impairment together with fluctuating cognition, parkinsonism and visual hallucination. To better understand brain abnormalities in DLB, numerous MRI studies have been performed but very few assessed resting-state functional connectivity, and only in a perspective of spatial analysis (Lowther et al., 2014; Peraza et al., 2014). Indeed, none of them focused on network time-courses or on interactions between networks. Yet, these are particularly relevant in DLB as cognitive fluctuations and hallucinations suggest that functional brain abnormalities in DLB may be transient. For computational concerns, and considering the number of volumes acquired per subject, we focused on seven specific RSNs exhibited during the resting-state fMRI session. They were chosen according to their relevance with respect to the disease, which is characterized by dysexecutive/attentional disorders (Ferman et al., 2006, 2013; Johns et al., 2009; Yoon et al., 2015), and visual (Ferman et al., 2006, 2013) and motor (McKeith et al., 2005) impairments:

- DMN (default mode network): self-oriented cognition,
- LFPN (left fronto-Parietal network): executive control,
- RFPN (right fronto-Parietal network): executive orientation of attention,
- OPFN (occipito-parieto-frontal network): visuo-oculomotor pathway, visuo-attentional and visuo-constructive processing,
- OPN (occipital posterior network): visual processing,
- MON (medial occipital network): visual processing,
- BG (basal ganglia): motor processing.

In assessing brain dynamic functional connectivity in DLB based on product HMM, our purpose is double: (1) to differentiate DLB patients from healthy elderly controls from their DFC analyses, and (2) to provide new analysis tools to explore DFC and characterize interactions between RSNs.

## 2. Materials and methods

### 2.1. Data

#### 2.1.1. Participants

Forty-four participants were recruited for this study from the Memory Resources and Research Center of the University Hospital of Strasbourg, France. Six participants were excluded due to excessive motion during the fMRI acquisition i.e., translation and rotation respectively higher than 2 mm or 2 degrees, according to the motion parameter resulting from SPM) and two participants were excluded due to neurological abnormalities. Therefore, groups consisted in 16 patients with DLB at the stage of mild dementia [8 females; mean age, 74.7 (range: 54–89) years] and 20 healthy elderly controls [11 females; mean age, 64.4 (range: 46–76) years]. All patients had formal assessment of their diagnosis by three independent expert clinicians. Controls underwent similar clinical, cognitive, psychiatric and neurological assessments to exclude any that may have had occult cognitive impairments. All patients satisfied McKeith's criteria for probable DLB i.e., at least two core symptoms out of three (McKeith et al., 2005). No patients shared both DLB and Alzheimer's disease clinical features. The MMSE (Mini Mental State Examination) scores (Folstein et al., 1975), as a cognitive functions scale, were 20.8 (std: 3.2; range 15–24) and 29.0 (std: 1.0; range 27–30) respectively, for patients and controls. Exclusion criteria included contraindications for MRI, history of alcohol/substance misuse, evidence suggesting alternative neurological or psychiatric explanations for their symptoms, focal brain lesions on brain imaging, and the presence of other severe or unstable medical illness. This study was approved by the local Ethics Committee (Comité de Protections des Personnes Est IV, Strasbourg, France). Controls and patients gave written informed consent. Patients were older than controls (two-sample *t*-test, *p* < 0.01), but did not differ in terms of gender (chi-squared tests at *p* < 0.05) (see Table S1 for details on the database).

#### 2.1.2. Data acquisition

A concomitant resting-state blood-oxygen-level-dependent (BOLD) and pulsed arterial-spin labeling (ASL) sequence was performed on a Siemens Verio 3T scanner equipped with a 32-channel head coil (Siemens, Erlangen, Germany). One-hundred twenty-one whole brain T2^*^-weighted (gradient echo) echo planar images were acquired using the QUIPPS II sequence provided by the manufacturer. Parameters were: TR = 3 s; flip angle = 90; TE = 21 ms; TI1 = 600 ms, TI2 = 1325.1 ms; FOV = 152 × 256 × 112 mm; imaging matrix: 38 × 64 × 28; 4 mm isotropic voxels, acceleration factor (generalized autocalibrating partially parallel acquisitions). The first volume served for ASL assessment and was therefore not considered for functional connectivity. A 3D MPRAGE T1-weighted image was also acquired at the same session. Parameters were: imaging matrix 192 × 192 × 176; 1 mm isotropic voxels.

### 2.2. Theory: Product HMM

A product HMM λ is a standard HMM built upon a set {λ^*k*^} of HMM, by taking into account their temporal interdependencies (Nefian et al., 2002). First, as a reminder, it should be noted that an HMM λ^*k*^ is a double stochastic process (*X*^*k*^, *Y*^*k*^) where $X^{k}=\left(X_{1}^{k},\cdots \;,X_{T}^{k}\right)$ is a hidden Markov chain observed through the observation sequence $Y^{k}=\left(Y_{1}^{k},\cdots \;,Y_{T}^{k}\right)$ of length *T*. $X_{t}^{k}=i^{k}$ means that λ^*k*^ is in state *i*^*k*^ at time *t*. *S*^*k*^ = {*i*^*k*^} is the state space of *X*^*k*^. The product HMM λ built upon {λ^*k*^}, 1 ≤ *k* ≤ *K*, is a double stochastic process (**X**, **Y**) in which state space *S* is by definition the Cartesian product of the *S*^*k*^, hence the term product HMM:

(1) $$\begin{array}{l} S≜S^{1}\times S^{2}\times \cdots \times S^{K} \end{array}$$

**X** = (**X**_1_, ⋯ , **X**_*T*_) is a hidden Markov chain where ${\text{X}}_{t}={\left(X_{t}^{1},\cdots \;,X_{t}^{K}\right)}^{\text{T}}$ denotes the state vector at *t*. **X**_*t*_ = **i** means that λ is in state **i** = (*i*^1^, ⋯ , *i*^*k*^, ⋯ , *i*^*K*^), **i** ∈ *S*. **Y** = (**Y**_1_, ⋯ , **Y**_*T*_) is the observation sequence of λ where ${\text{Y}}_{t}={\left(Y_{t}^{1},\cdots \;,Y_{t}^{K}\right)}^{\text{T}}$ is obtained through the concatenation of the observations $Y_{t}^{k}$. The parameters of a product HMM λ are:

(2) $$\begin{array}{ll} \pi_{\text{i}} & =P\left[{\text{X}}_{1}=\text{i}\right], \end{array}$$

(3) $$\begin{array}{ll} a_{\text{i}\text{j}} & =P\left[{\text{X}}_{t}=\text{j}|{\text{X}}_{t-1}=\text{i}\right], \end{array}$$

(4) $$\begin{array}{ll} b_{\text{j}}\left({\text{Y}}_{t}\right) & =P\left[{\text{Y}}_{t}|{\text{X}}_{t}=\text{j}\right] \end{array}$$

Π = {π_**i**_, **i** ∈ *S*}, is the set of initial probabilities where π_**i**_ denotes the probability to be in state **i** at time *t* = 1. The transition matrix *A* = {*a*_**ij**_, **i**, **j** ∈ *S*}, reflects the temporal dependencies between the *K* hidden Markov chains. It probabilistically models their joint state evolution in time. *B* = {*b*_**j**_(·), **j** ∈ *S*}, is the set of observation probabilities associated with states **j**. In practice, the conditional independence of $Y_{t}^{k}$ given $X_{t}^{k}$ is assumed, in Equation (4), so as:

(5) $$\begin{array}{l} P\left[{\text{Y}}_{t}|{\text{X}}_{t}=\text{j}\right]=\underset{k}{\prod }P\left[Y_{t}^{k}|X_{t}^{k}=j^{k}\right]=\underset{k}{\prod }b_{j^{k}}\left(Y_{t}^{k}\right) \end{array}$$

**Figure 1:**
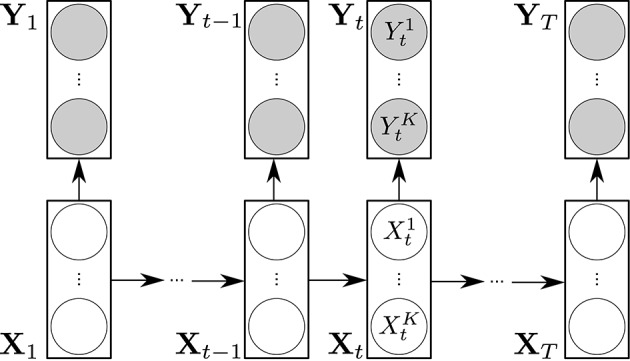
**Representation of a product HMM as a dynamic Bayesian network**. The transparent circles represent the hidden variables $X_{t}^{k}$. The shaded circles represent the observations $Y_{t}^{k}$. The rectangular boxes and their conditional dependencies represented by arrows depict the DBN representation of a standard HMM.

The dynamic Bayesian network representation of a product HMM is shown Figure 1.

As a standard HMM, a product HMM preserves the algorithmic aspects of an HMM related to the evaluation, learning and decoding procedures (Rabiner, 1989; Nefian et al., 2002). The likelihood *P*(**Y**|λ) can be calculated to assess how well the model fits the observed data and vice versa (*evaluation*). The product HMM parameter set θ = {π_**i**_, *a*_**ij**_, *b_j^k^_*(▪); **i**, **j**, *j*^*k*^ ∈ *S*^*k*^, 1 ≤ *k* ≤ *K*} can be learned by iteratively training the model λ(θ) to produce **Y** (*learning*). The optimal, *K*-dimensional state sequence $\hat{\text{X}}$, and the resulting *K* state sequences ${\hat{X}}^{k}$, underlying **Y** can be inferred or decoded to lead to the “symbolic” state-based transcription of **Y** (*decoding*). Finally, product HMM can be compared in pairs using the distance measure between two models λ_1_ and λ_2_, defined in Juang and Rabiner (1985) and Rabiner (1989) as:

(6) $$\begin{array}{l} D\left(\lambda_{1},\lambda_{2}\right)=\frac{1}{T}\left[\log P\left({\text{Y}}_{\left(2\right)}|\lambda_{1}\right)-\log P\left({\text{Y}}_{\left(2\right)}|\lambda_{2}\right)\right] \end{array}$$

where **Y**_(2)_ is a sequence of observations generated by model λ_2_. Equation (6) is a measure of how well λ_1_ matches observations generated by λ_2_, relative to how well λ_2_ matches observations generated by itself. Several interpretations of Equation (6) exist in terms of cross entropy, divergence or discrimination information (Juang and Rabiner, 1985). The symmetrized version of this measure is

(7) $$\begin{array}{l} D_{1-2}=\frac{D\left(\lambda_{1},\lambda_{2}\right)+D\left(\lambda_{2},\lambda_{1}\right)}{2} \end{array}$$

Further details of product HMM can be found in Nefian et al. (2002).

### 2.3. Product HMM for dynamic functional analysis of RSNs

#### 2.3.1. Data preprocessing and organization

As the TE is high enough to make the BOLD sequence sensitive to ASL signals, data were low-pass filtered at 0.1125 Hz according to the method of Chuang et al. (2008) to remove ASL frequencies. After this filtering, each subject underwent the following preprocessing steps using the SPM8 toolbox (Statistical Parametric Mapping, 2009): slice-timing correction; rigid body registration with correction of effects of B0 field inhomogeneities; coregistration to anatomical space; spatial normalization onto the MNI space with the DARTEL approach i.e., from the transformation parameters provided by the T1-weighted image normalization).

A group-level spatial independent component analysis (ICA) was carried out using the GIFT toolbox (GIFT, 2004) to extract the common RSNs of all the subjects (controls and patients included). We used 80 components for the subject-specific data reduction with principal components analysis (PCA) according to the automatic estimator available in GIFT, and 30 components for the group data reduction. The ICA with Infomax algorithm (Bell and Sejnowski, 1995) was repeated 10 times using ICASSO (Himberg et al., 2004) to provide stable components.

**Figure 2:**
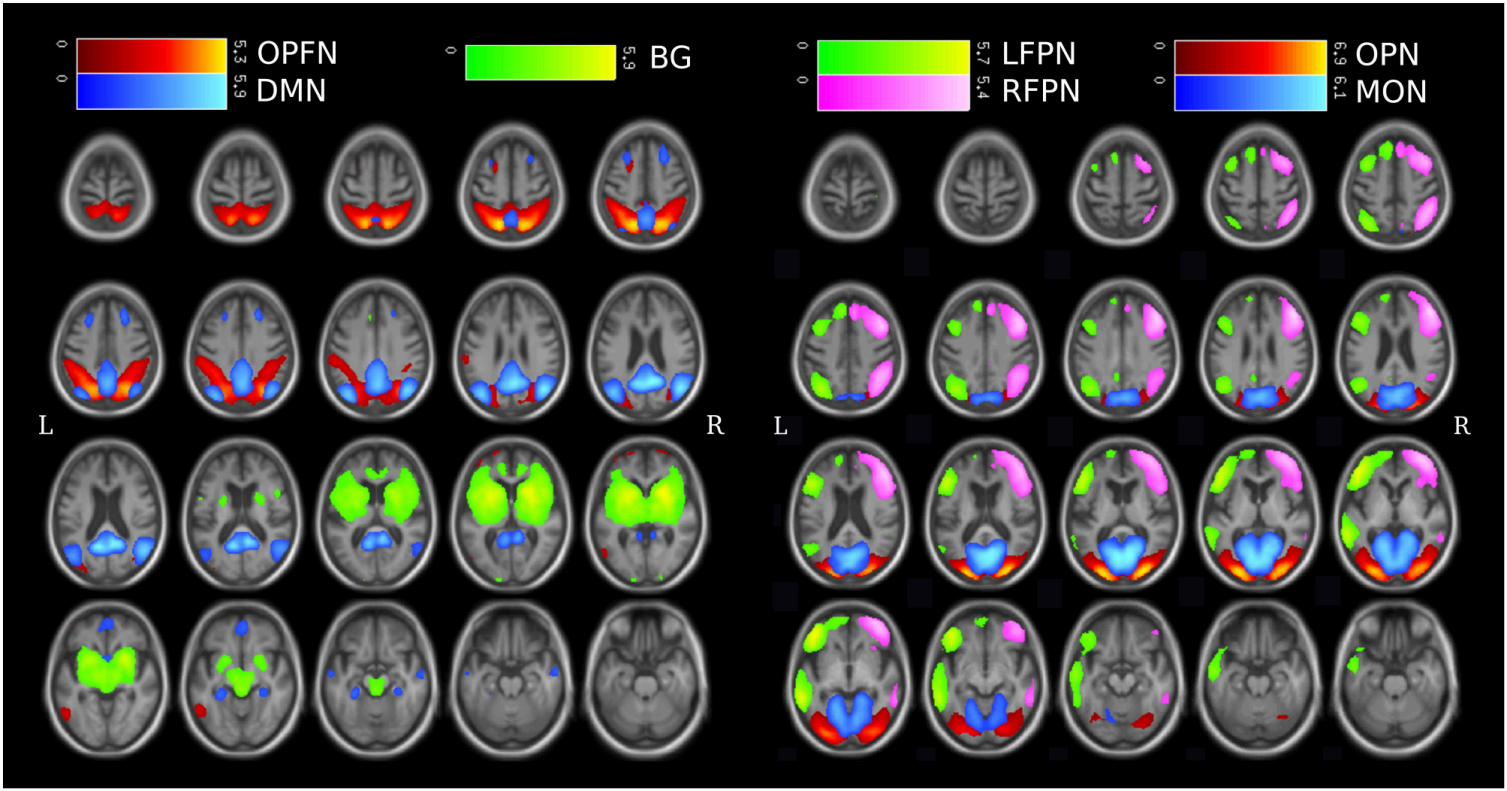
**Spatial maps of the 7 studied RSNs from group-ICA expressed in z-score, plotted upon mean T1 from all subjects (patients and controls combined)**. (DMN, Default Mode Network; LFPN, Left Fronto-Parietal Network; RFPN, Right Fronto-Parietal Network; OPFN, Occipito-Parieto-Frontal Network; OPN, Occipital Posterior Network; MON, Medial Occipital Network; BG, Basal ganglia.)

According to previous RSN templates from group ICA (Damoiseaux et al., 2006), two experts selected among the 30 components the ten that match a RSN. For computational concerns, and considering the number of volumes acquired by subject, we focused on seven specific RSNs (see Figure 2) described in Section 1: the default mode network, the left fronto-parietal network, the right fronto-parietal network, the occipito-parieto-frontal network, the occipital posterior network, the medial occipital network and the basal ganglia. These networks were relevant due to their possible implications in the DLB disorders, such as attention and executive functions (the fronto-parietal networks), visual processing (the occipital networks), parkinsonism (the basal ganglia network), or for being a major RSN (the default mode network). The three remaining RSNs consisted in a frontal network and two central networks (see Figure S1 for the spatial maps of the frontal network and a central network).

For each of these RSNs, a subject-specific spatial map and its associated time-course were back-reconstructed (Calhoun et al., 2001). It led to 7 × 16 = 112 time-courses for the patients group and 7 × 20 = 140 time-courses for the control group.

**Figure 3:**
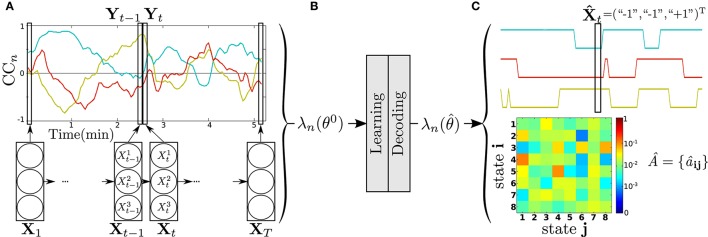
**DFC modeling and analysis steps of a subject, for RSN *n* vs. the others with *N* = 4, *K* = 3 (see text)**. **(A)** Product HMM modeling of the corresponding correlation plot CC_*n*_. **(B)** Parameter initialization, learning, decoding. **(C)** Two examples of output results: decoded state sequence $\hat{\text{X}}$ (top), transition matrix Â estimated by learning (bottom).

Then, to reduce the parameters set θ to learn per model λ, RSN data of each subject were organized according to a “one RSN *vs*. the others” analysis strategy. For each RSN *n*, 1 ≤ *n* ≤ *N* = 7, a correlation coefficient plot CC_*n*_ = {cc_*n, m*_(*t*); 1 ≤ *m* ≤ *N* = 7, *m* ≠ *n*, 1 ≤ *t* ≤ *T*} was formed. It is composed of *K* = *N* − 1 = 6 time series cc_*n, m*_(*t*) of length *T* representing the evolution of the correlation coefficient for each pair of time-courses associated with RSN_*n*_ and RSN_*m*≠*n*_. cc_*n, m*_(*t*) was calculated using a sliding tapered window made by convolving a Gaussian with a rectangle of 15^*^TR (45 s) and sliding in steps of 1 TR, leading to *T* = 103 windows. An example of CC_*n*_ is shown at the top of Figure 3A, for *N* = 4 (*K* = 3) for clarity.

#### 2.3.2. DFC modeling and analysis by product HMM

The method was implemented with Matlab R2012b (The Mathworks Inc., Natick, MA, USA). The DFC modeling procedure is illustrated in Figure 3A. A model λ_*n*_ is assigned to each CC_*n*_, leading to 7 product HMM λ_*n*_ per subject. The correlation coefficient plot CC_*n*_ is considered as the observable process of λ_*n*_, that is, **Y** ≡ CC_*n*_. $\text{Y}=\left\{Y_{t}^{k}\right\}$ is thus a *K* × *T* matrix of observations with $Y_{t}^{k}={\text{cc}}_{n,m}\left(t\right)$. As described in Section 2.2, the product HMM λ_*n*_ is built upon 6 HMM $\lambda_{n}^{k}$ with 1 ≤ *k* ≤ *K* = 6. $\lambda_{n}^{k}$ models the *k*^th^ correlation coefficient time series, or row, of **Y**. The state space *S*^*k*^ of $\lambda_{n}^{k}$ is composed of two states, the anticorrelation state “−1” and the correlation state “+1,” so that the state space of λ_*n*_ is *S* = {“−1,” “+1”}^*K*^, with Card(*S*) = 2^6^ = 64. The transition matrix *A* = {*a*_**ij**_, **i**} and **j** in *S*, models the temporal dependencies between the correlation coefficient times series of **Y**. The matrix *A* of dimension 64 × 64, captures the overall dynamics of the CC_*n*_. The conditional probability density functions (pdf) *b_j^k^_*(▪) model the distribution of $Y_{t}^{k}={\text{cc}}_{n,m}\left(t\right)$ given the state $X_{t}^{k}=j^{k}$, *j^k^* ∈ {“−1”;“+1”}. These pdf are assumed to be unimodal Gaussians with means $\mu_{j^{k}}$ and variances $\sigma_{j^{k}}$, so as Equation (5) can be written as:

(8) $$\begin{array}{l} P\left[{\text{Y}}_{t}|{\text{X}}_{t}=\text{j}\right]=\underset{k}{\prod }b_{j^{k}}\left(Y_{t}^{k}\right)=\overset{K}{\underset{k=1}{\prod }}\left[N\left(Y_{t}^{\left(k\right)},\mu_{j^{k}},\sigma_{j^{k}}\right)\right] \end{array}$$

As described in Figure 3B, before learning, the parameters of the model λ_*n*_ are initialized (θ = θ^0^). The initial probabilities of Equation (2) are set uniform, with $\pi_{i^{k}}=\frac{1}{64}$. Regarding to Equation (3), {*a*_**ij**_} have been set to $\frac{1}{64+1}$ except for the diagonal terms {*a*_**ii**_} set to $\frac{2}{64+1}$. The means $\mu_{j^{k}}$ and variances $\sigma_{j^{k}}$ were initialized directly from the data. For each channel *k*, we used the positive values of the correlation coefficients cc_*n, m*_(*t*) to calculate $\mu_{{}^{\prime \prime }+1^{\prime \prime }}$ and $\sigma_{{}^{\prime \prime }+1^{\prime \prime }}$ and the negative values for $\mu_{{}^{\prime \prime }-1^{\prime \prime }}$ and $\sigma_{{}^{\prime \prime }-1^{\prime \prime }}$. Then *b*_**j**_(**Y**_*t*_) are initialized as directly observable using Equation (8). Then, λ_*n*_ is iteratively trained to produce **Y** = CC_*n*_ until convergence $\left(\theta =\hat{\theta }\right)$. For computational concerns, and to keep the approach as simple as possible, without losing performance in decoding, the means and variances were not learned, but just initialized. After learning, the DFC model $\lambda_{n}\left(\hat{\theta }\right)$ is used to decode **Y**, i.e., to infer the hidden state sequence $\hat{\text{X}}$ that best explains the observation CC_*n*_. By way of example, Figure 3C shows the hidden state sequence $\hat{\text{X}}$ decoded from the CC_*n*_ of Figure 3A, as well as the transition matrix Â obtained after learning.

Finally, the DFC was compared between participants to detect if there was a significant DFC change between controls and patients. To this end, the DFC models λ_*n*_, 1 ≤ *n* ≤ 7, were compared in pairs by means of the distance measure *D* (see Equation (7)). First, the models were asked to generate *T*_*gen*_ = 150 then *T*_*gen*_ = 300 observations (against *T* = 103 for the real observations). Let {*D*_*c*−*c*_} be the distances between all pairs of controls (190 pairs) and {*D*_*c*−*p*_} the distances between all pairs of patients and controls (320 pairs). A two samples *t*-test was performed between the distance sets {*D*_*c*−*c*_} and {*D*_*c*−*p*_} for each of the 7 studied RSN. A positive result at a *t*-test (*p* < 0.01) would signify that a significant DFC change is observed between patients and controls with regard to the interactions between the RSN under concern and the six others. A complementary study has also been conducted that includes the other networks revealed by ICA (the central and the frontal networks), in place of BG and DMN which are less affected by DLB as previously described.

#### 2.3.3. Product HMM tools for DFC analysis

An interesting feature of product HMM, which makes the product HMM modeling and analysis framework attractive, is the range of analysis tools available for the user to investigate different aspects of the DFC. These tools can provide useful information of a global nature about the DFC or enable more insight to be gained into it. We have added more information about these tools and the interpretation of their output results before the Results section.

The distance measure of Equation (7) provides global information on the degree of similarity of two models of DFC: it allows pairs of models to be compared through their ability to separately generate the same observation sequence. We use this distance extensively to infer significant differences between the DFC models of DLB patients and those of healthy elderly subjects.

Also, the transition matrix *A* of a DFC model provides a compact and global representation of the statistical behavior of the observed DFC. It allows us to see at a glance which transitions are privileged, ignored or modified for a given subject or between subjects. But the transition matrix *A* also enables us to focus on more specific information. In particular, the diagonal term {*a*_**ii**_} corresponds to the loopback probability of state **i**, that is, the probability to stay in state **i** from *t* to *t* + 1. Its magnitude reflects the temporal stability of the DFC state or DFC configuration **i**. A brief look at the diagonal of *A* thus enables the number and nature (correlated or uncorrelated resting-state network involved) of steady DFC configurations to be rapidly identified. Similarly, the presence of high transition probabilities {*a*_**ij**_} on a column **j** in *A* is symptomatic of a DFC state, in this case state **j**, with a high probability to be reached. We therefore used the average of the terms of this column, $\underset{\text{j}}{\sum }a_{\text{i}\text{j}}$ to quantify this probability.

The N-dimensional decoded state sequence $\hat{\text{X}}$ gives access to the symbolic transcription, expressed as a visited states-time sequence, of what is observed, in our case the correlation over time of one RSN with other RSNs. By preserving time information, the sequence $\hat{\text{X}}$ enables analysis to be focused on specific temporal windows of the fMRI exam. Such sequences can also be the starting point for simple or advanced analyses that enrich the DFC description. These analyses can be performed on a single sequence $\hat{\text{X}}$, becoming specific of $\hat{\text{X}}$, or performed on a set {$\hat{\text{X}}$} of decoded state sequences, in relation to a subject, a particular RSN, a group of subjects, or whatever the user wishes to target. It is thus possible to perform simple statistics that highlight the most common states, the ones never reached, the time spent in each state or the number of transitions. Advanced analyses, outside the scope of this paper and based on pattern recognition or data mining techniques, can also be envisaged to detect, for example, reproducible patterns of DFC.

## 3. Results

### 3.1. Distances between controls and DLB patients

Table 1 shows the results of the two-sample *t*-tests for each of the seven RSNs, for *T*_*gen*_ = 150 and *T*_*gen*_ = 300 observations generated (see Equation 6). The occipito-parieto-frontal network (OPFN), the medial occipital network (MON) and the right fronto-parietal network (RFPN) presented a highly significant difference (*p* < 0.01) in DFC between patients and controls, for both *T*_*gen*_. (see Table S2 for the results for the complementary study with the frontal and central networks).

**Table 1:** ***p*-value of the *t*-tests for each RSN and number of generated observations**.

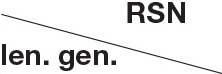	**OPFN**	**MON**	**RFPN**	**LFPN**	**OPN**	**DMN**	**BG**
150	**1.10^−4^**	**1.10^−9^**	**1.10^−3^**	0.324	0.266	0.200	0.870
300	**1.10^−5^**	**1.10^−10^**	**1.10^−5^**	0.026	0.400	0.049	0.794
*h*	**1**	**1**	**1**	0	0	0	0

*h = 1 indicates significant difference between DFC models of patients vs. controls (p < 0.01). DMN, Default Mode Network; LFPN, Left Fronto-Parietal Network; RFPN, Right Fronto-Parietal Network; OPFN, Occipito-Parieto-Frontal Network; OPN, Occipital Posterior Network; MON, Medial Occipital Network; BG, Basal ganglia*.

### 3.2. Product HMM analysis tools

We present selected product HMM results obtained for the OPFN, MON, and RFPN. They illustrate how to obtain deeper insight into the DFC difference observed between DLB patients and controls for these three RSNs.

#### 3.2.1. Transition matrices

For all participants (patients and controls), a close examination of the transition matrices of these networks shows that the states with the highest probability to be reached are those with the OPN, the OPFN, and/or the MON in a “correlated” state (“+1”). A comparison of the DLB patients' transition matrices with the healthy subjects' transition matrices significantly revealed that states with MON and RFPN in a “correlated” state were more probably reached for healthy subjects than for patients (*p* = 0.044 < 0.05).

**Figure 4:**
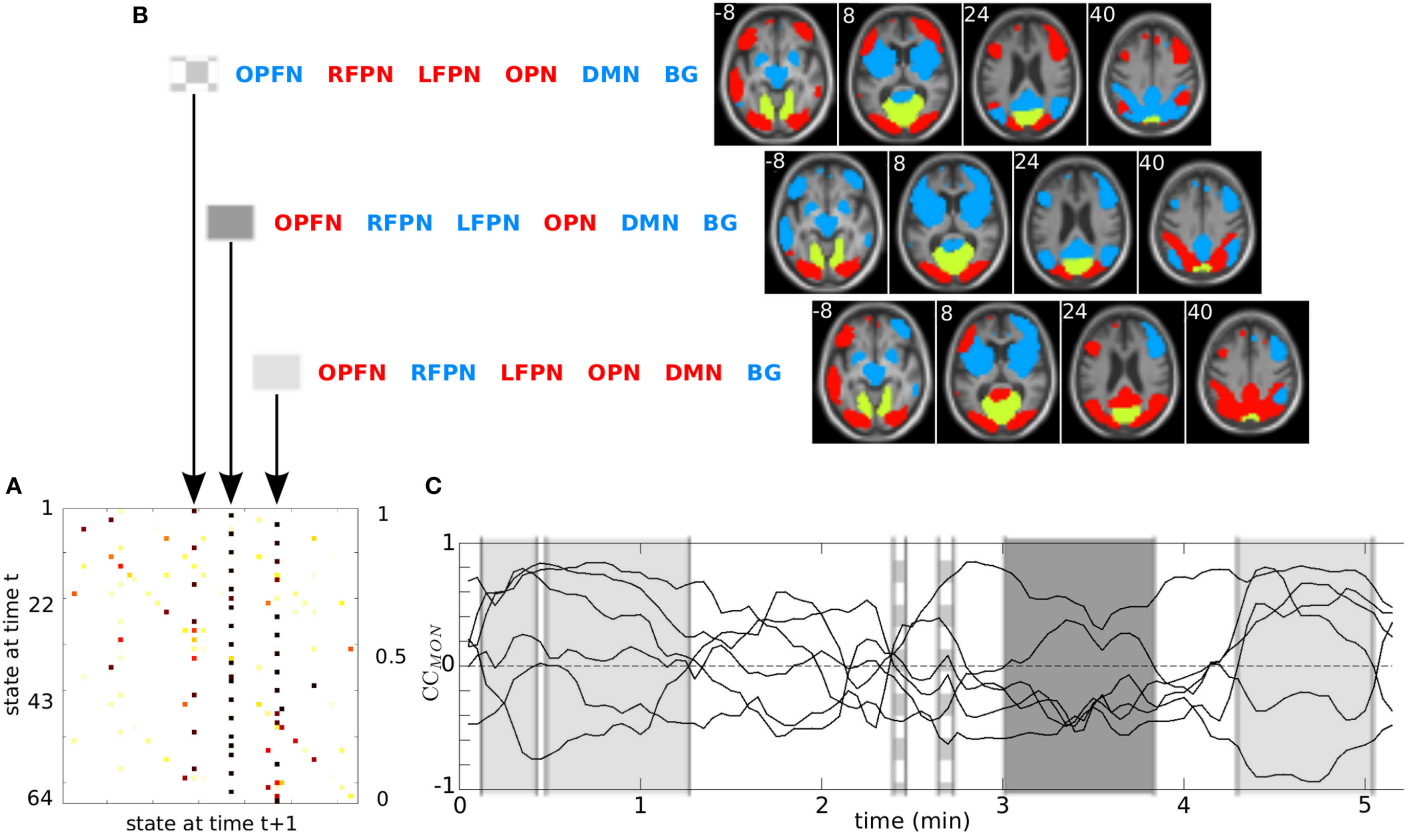
**Outputs of the product HMM for patient #1: (A) transition matrix *A*; (B) the 3 states with highest probabilities (coordinates in z axis are in white); (C) distribution of these 3 states reported on the CC_MON_**. On this figure, the MON is the reference RSN (in yellow on the RSN maps). (DMN, Default Mode Network; LFPN, Left Fronto-Parietal Network; RFPN, Right Fronto-Parietal Network; OPFN, Occipito-Parieto-Frontal Network; OPN, Occipital Posterior Network; MON, Medial Occipital Network; BG, Basal ganglia.)

Other typical product HMM output results are presented in Figure 4A, for a patient, and for the MON. The transition matrix *A* allowed us to isolate three states with the highest probability to be reached. These are, from left to right, the {opfn-RFPN-LFPN-OPN-dmn-bg}, the {OPFN-rfpn-lfpn-OPN-dmn-bg} and the {OPFN-rfpn-LFPN-OPN-DMN-bg} (with “correlated” states in upper case letters and “anti-correlated” states in lower case). The spatial maps of the “correlated” RSNs (in red) and the “anti-correlated” RSNs (in blue) with the MON (in yellow) are displayed in Figure 4B.

#### 3.2.2. Decoded state sequences

From the decoded state sequences ${\hat{\text{X}}}_{\text{OPFN}}$, ${\hat{\text{X}}}_{\text{MON}}$, and ${\hat{\text{X}}}_{\text{RFPN}}$, no difference was observed in the state change statistics. Twenty-two state changes and 18 distinct states were observed on average for each session of 6 min. On average, 3 to 4 states per subject, replicated at different times, presented a “long” state occupancy duration i.e., greater than one time-point). These states varied across subjects, even if we noticed that the most visited state (long and replicated) for ${\hat{\text{X}}}_{\text{OPFN}}$ and for ${\hat{\text{X}}}_{\text{MON}}$ was the same for both patients and controls, namely the state {OPFN/MON-rfpn-lfpn-OPN-DMN-bg}, (with “correlated” states in upper case letters and “anti-correlated” states in lower case). The mean of the state occupancy duration was 14 s for both DLB patients and controls (see Figure S3).

Figure 4C shows a typical example of targeted information that can be derived at the subject level from a decoded state sequence: the distribution of three selected states, with their duration, along the time axis of the session. The three highlighted states are {opfn-RFPN-LFON-OPN-dmn-bg}, {OPFN-rfpn-lfpn-OPN-dmn-bg}, and {OPFN-rfpn-LFPN-OPN-DMN-bg}. Figure S2 presents the decoded state sequences ${\hat{X}}^{k}$ of each pair of interactions. Figure S4 zooms in on the first 30 s of the observation and decoded sequences that are presented Figure 4C.

#### 3.2.3. Combining product HMM results

Based on the transition matrices of the MON, a relationship between this visual network and the attentional network RFPN was first underlined in Section 3.2.1. The following analysis shows how combining different product HMM results can provide extra information about specific RSN interactions. Focusing on interactions between the MON and the attentional networks (RFPN, LFPN) indeed showed that the sequences are significantly more occupied by states with “correlated LFPN,” “anti-correlated RFPN” (whatever the correlation state, “−1” or “+1,” of the other RSNs) for patients than for controls (*p* = 0.021 < 0.05, Kruskal–Wallis non-parametric test). The mean occupation rate in a sequence by these states was 40% for patients against 22% for controls.

## 4. Discussion

### 4.1. DLB characterization

Compared to healthy elderly controls, patients with DLB showed changes in the dynamic interaction between the MON and the other networks, so did the OPFN and the RFPN. According to previous studies, the MON concerns visual processing (Beckmann et al., 2005), the OPFN underlies visuo-attentional and visuo-constructive processing (Fox et al., 2006), and the RFPN relates to executive control of attention (Seeley et al., 2007; Markett et al., 2014). Since patients with DLB suffer mainly from attentional (Ferman et al., 2006; Johns et al., 2009; Ferman et al., 2013; Yoon et al., 2015) and visual (Ferman et al., 2006, 2013) impairments, the changes of DFC of these three networks are therefore consistent with the cognitive profile of DLB. They are also consistent with previous MRI studies on brain perfusion (Lobotesis et al., 2001; Colloby et al., 2002) and metabolism (Kantarci et al., 2012), which have reported a decrease of neural activity in the occipital cortex and frontal and parietal areas. Despite the occipital cortex having been suggested as the main deficit in DLB, static functional connectivity (Lowther et al., 2014; Peraza et al., 2014) failed to reveal any difference in visual networks, in contrast to our dynamic functional connectivity analysis. In addition, results from the transition matrix revealed that states involving “correlated” MON and RFPN, whatever the other networks, are significantly less likely to be reached in patients than in controls. This suggests a lack of specific interaction between right top-down attentional and bottom-up visual processes. On a similar note, focusing on the occupancy rate of states involving “correlated” MON and LFPN and “anti-correlated” RFPN highlighted a significant increase for DLB patients compared to controls. A similar trend for these states was observed when the OPFN is taken as the reference RSN instead of the MON. Taken together, all these results of DFC suggest that, in DLB, the visual networks and particularly the MON might interact less with the RFPN than with the LFPN. Such a result could reflect a compensatory phenomenon for the deficit of right attentional and visual interaction.

Whereas previously identified differences concerned the global dynamics, they are not apparent from the number of transitions, the average duration in a state or the number of visited states. These measurements still provide information about variability of functional connectivity during an fMRI session. Thus, on average, only 18 distinct states were visited among the 64 theoretically observable, during a session of 6 min, with an average stability in a state of 14 s. Still, one might raise the question of the influence of age on this DFC characterization.

In addition, the presented method is performed at subject level. This allows the DFC study to be to individualized and can be useful for individual monitoring purposes. For instance, Figure 4 illustrates this possibility with patient 1. Unsurprisingly, the states reached with the highest probabilities are those with the MON, the OPFN, and the OPN, the three visual networks, working together. Examining each decoded sequence, ${\hat{X}}^{k}$, related to the PHMM_*MON*_, we noticed that the LFPN and the RFPN seemed to appear mostly in opposite timing (see Figure S2), with quasi-synchronous changes of state. This might be interpreted from the perspective of inter-hemispheric valence (Banich, 1998) and informs us on the dissociation of these two networks that are, however, very related from an functional connectivity point of view. Now that specific RSNs (OPFN, MON, RFPN) have shown their interest in DFC for DLB studies, a PHMM-based model, built on a chosen network for each DLB patient, can be considered as a new application for specific DFC characterization of a resting-state network.

### 4.2. Product HMM modeling and analysis of DFC

All the presented results show the capabilities of product HMM to model and analyze multiple processes interacting in time, such as the DFC between networks observed by resting-state fMRI. They also demonstrate how it is possible to capture by statistical learning, spatial and temporal relationships between RSNs within a probabilistic state graph. Finally, they illustrate the range of analysis tools that are immediately available within the product HMM framework i.e., to compare distinct DFC from their model, or to further characterize them from their state transition matrix or from their decoded state sequence.

From a modeling perspective, we chose a multi-dimensional HMM to represent the processes of the RSNs and thus we defined the states as vectors of sub-states. Our study, although providing tools to differentiate DLB patients from healthy controls, was also designed to present a method to explore DFC. To this end, the states are built on the RSNs present during the session, and prior to the DFC analysis. A large state space is thus constructed and accounts for all possible multidimensional connectivity states between RSNs, even if most of them are not, or cannot be, observed within the limited time windows of an resting-state fMRI session. This leads to a high number of states compared to the one defined directly on the DFC observations as DFC analysis methods based on state clustering (Allen et al., 2014; Eavani et al., 2013; Ma et al., 2014). While it is true that a large state space can lead to a certain redundancy due to close spatial profiles (functional connectivity maps of states) between states, this allows the emergence of finer connectivity configurations, including states with short duration and low occurrence. Note that these close spatial profiles can be *a posteriori* gathered to restrict the analysis to some chosen states if one wants to target some RSNs (an illustration can be found in Figure S2 where similar color tones have been applied to highlight specific interactions). This allows a better understanding while ensuring that a larger number of RSNs had been taken into account during the modeling step.

Still from a modeling perspective, the proposed approach remains simple yet efficient. It is of low complexity, and presents some limitations. It is simple regarding the state sub-space *S*^*k*^ since we did not consider a decorrelated situation between two RSNs, as might be done with a “0” state in addition to the “−1” and “+1.” A direct consequence of this modeling extension would be a transition matrix *A* of dimension 3^*K*^ × 3^*K*^. The introduction of such a neutral state also raises the question of the threshold above and below which we consider, respectively, the correlated and uncorrelated state. DFC modeling is not well known and a question rises as to whether or not the DFC is modulated by a central executive. Exploiting a hierarchical structure would enable modeling different levels and length scales that might be present in the brain DFC. Our approach also relies on a compromise between the state space cardinality and the available amount of data. First, we only capture what may be seen throughout the duration of the fMRI session. On the other hand, the parameter space grows exponentially with the number of hidden processes, leading us to limit the approach to the joint analysis of a subset of RSNs. Although our results finally showed that only a few states were achieved with regard to the possibilities (about 18 distinct states observed within the 64 defined and 103 time-points), too many states would have meant that the parameter estimation step would not have been robust. Possible solutions to these issues could be to reduce the number of conditional dependencies between states, as proposed in coupled Markov models, another variant of multi-dimensional HMM, or to extensively use parameter tying in order to limit drastically the total number of parameters to be learned.

From an analytic perspective, the product HMM tools are numerous. The transition matrix provides information on the most likely transitions, on the state with the highest probability to be reached or on the stability of states, expressed in terms of probability with the values of the diagonals {*a*_**ii**_}. The decoded state sequence brings information at different scales of analysis : at a global scale when considering $\hat{\text{X}}$, at a lower scale when looking for specific interactions with ${\hat{X}}^{k}$. On one hand, $\hat{\text{X}}$ gives us access to the number of state changes, the most reproduced states, the average duration in a state and especially the temporal distribution of the states in the session. This latter aspect is of particular interest if one seeks to interpret certain functional connectivity states only present at the beginning or at the end of the session as, for example, in resting-state studies, or in task-based fMRI. On the other hand, the ${\hat{X}}^{k}$ focuses on a particular interaction. For instance, the ratio between the number of “correlated” and “anti-correlated” states offers information about the relationship between two specific RSNs.

Product HMM, of course, do not and will not reveal all the aspects of DFC. However, we believe that such advanced graph theoretical approaches open the door to complementary analysis techniques, of a higher level and working on top of product HMM, such as data mining, structural analysis, pattern recognition, chronic extraction, and/or temporal reasoning. These techniques, coupled with product HMM, should help us to answer typical questions in DFC analysis, such as: which RSNs, at what time and for what duration enter in correlation? Does the DFC pattern selected by the user exist, and if so, is it reproducible, and to what extent? Does any significant DFC pattern emerge across this pool of fMRI data? More broadly, these techniques applied to product HMM outputs should reveal not only DFC patterns but also “DFC grammars” subject-, group-, or disease-specific in relation, for this latter aspect, with the concept of biomarker.

## Conclusion

We presented a novel graph theoretical approach for the modeling and analysis of brain dynamic functional connectivity. The approach is based on product HMM, an instance of dynamic Bayesian network able to learn temporal dependencies between interacting processes. From the DFC modeling of a pool of RSNs, the product HMM framework was illustrated in differentiating patients with DLB from healthy elderly subjects. The comparison of distances between DFC models pointed out three RSNs serving the cognitive functions that are known to be impaired in DLB: mainly visual processing but also attentional and executive functions. Whereas static functional connectivity did not reveal any difference in visual networks in DLB, product HMM-based analysis of DFC succeeded in highlighting such occipital functional dysconnectivity. Novel statistical tools for further analysis of DFC were also presented within the product HMM framework. Their output results, even if some are difficult to interpret today, demonstrated the relevance of graph-based DFC modeling for the analysis and characterization of neurodegenerative diseases such as DLB. Also, they allow us to envisage in the near future the coupling of product HMM/DBN with data mining techniques and/or structural analysis techniques of a higher level to reveal DFC patterns or DFC “grammars” in relation to the concept of biomarker.

## Author contributions

MS and LT designed the study. DR performed MRI preprocessing. The data were analyzed by MS, LT, and DR and interpreted by MS, LT, DR, JF, and FB. Drafting of manuscript was done by MS, LT and DR and LT, DR, JA, JF, and FB critically reviewed the study proposal.

## Funding

This study was funded by Projet Hospitalier de Recherche Clinique (PHRC) inter-régional (IDRCB 2012-A00992-41) and supported by the imaging facility “Imagines” of ICube laboratory, UMR 7357, FMTS, University of Strasbourg.

### Conflict of interest statement

The authors declare that the research was conducted in the absence of any commercial or financial relationships that could be construed as a potential conflict of interest.
