# Supplementary material for: Identifying Dynamic Functional Connectivity Changes in Dementia with Lewy Bodies Based on Product Hidden Markov Models
Source: Front Comput Neurosci. 2016 Jun 23;10:60. doi: 10.3389/fncom.2016.00060 (PMC4918689; doi:10.3389/fncom.2016.00060)
Supplement: Supplementary file 1 [file Presentation1.pdf]

Supplementary Material: Identifying dynamic functional connectivity changes in dementia with Lewy bodies based on product hidden Markov models

Marion Sourty [1,*], Laurent Thoraval [1], Daniel Roquet [1], Jean-Paul Armspach [1], Jack Foucher [1,2] and Frédéric Blanc [1,3]

*Correspondence:
Marion Sourty
marion.sourty@icube.unistra.fr

1 SUPPLEMENTARY TABLE

Table S1. Demographic table: mild-DLB, dementia with Lewy bodies at the stage of dementia; HC, healthy controls. MMSE: mini mental state examination. std: standard deviation.

**Characteristic**	**mild-DLB**	**HC**
Participants	16	20
Female	8	11
Age, years (std)	74.7 (10.2) *	64.4 (8.6)
MMSE score (std) (max 30)	20.8 (3.2)	29.0 (1.0)
Education, years (std)	7.9 (3.0)	13.6 (2.5) *
Participants with visual hallucinations	9	0
Participants with fluctuations	8	0
Participants with parkinsonism	10	0

*, $p < 0.01$, compared to HC resulting from two sample t-tests.

Table S2. p-value of the t-tests for each RSN and number of generated observations. h = 1 indicates significant difference between DFC models of patients *vs.* controls (p<0.01).

RSN / len. gen.	OPFN	MON	RFPN	LFPN	OPN	FN	CN
150	**2.10^{-4}**	**1.10^{-8}**	**1.10^{-4}**	0.012	0.657	0.606	0.065
300	**0.004**	**1.10^{-7}**	**3.10^{-4}**	0.172	0.426	0.763	0.426
h	**1**	**1**	**1**	0	0	0	0

DMN: default mode network; LFPN: left fronto-parietal network; RFPN: right fronto-parietal network; OPFN: occipito-parieto-frontal network; OPN: occipital posterior network; MON: medial occipital network; BG: basal ganglia

2 SUPPLEMENTARY FIGURES

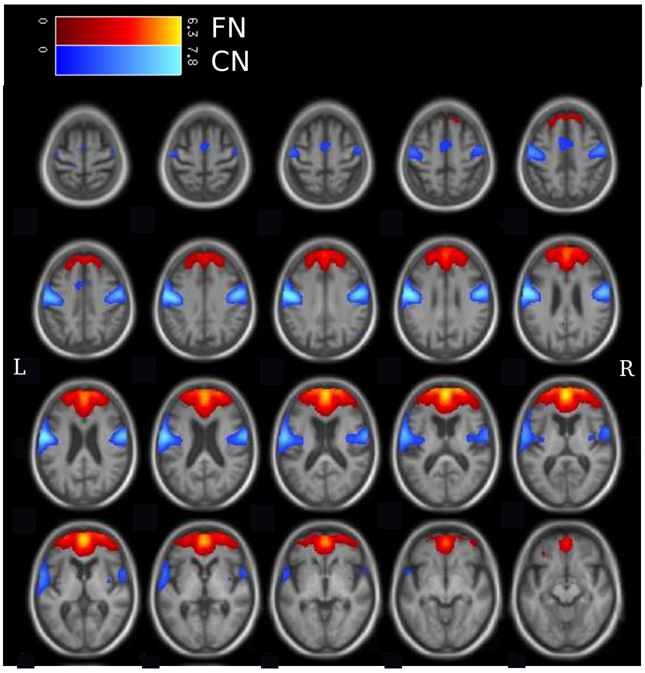

Figure S1. Spatial maps of the RSNs studied for the complementary analysis presented Table S2. Extracted from group-ICA expressed in z-score, plotted upon mean T1 from all subjects (patients and controls combined). (CN: central network; FN: frontal network).

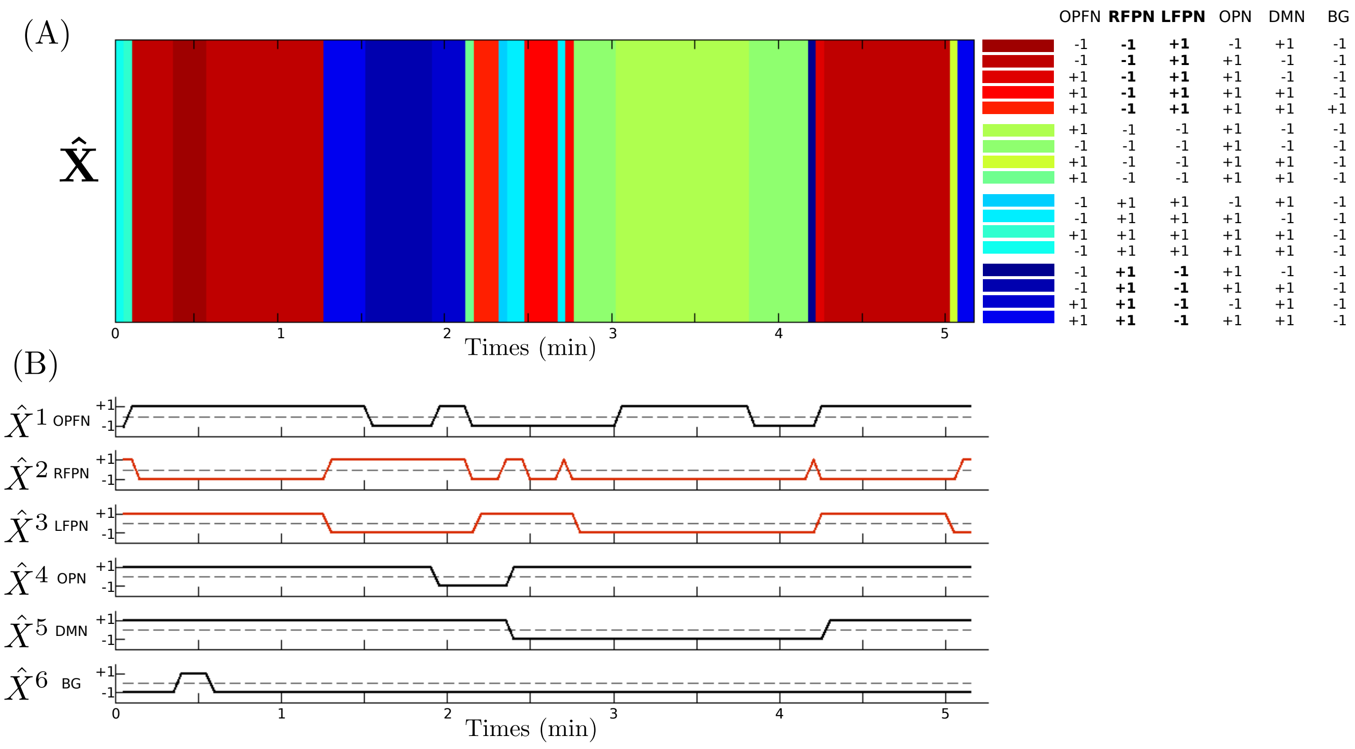

Figure S2. Decoded sequences $\hat{\mathbf{X}}$ and $\hat{X}^k$ of PHMM$_{\mathrm{MON}}$ for patient 1. In (A), the states are classified by colors: red tones for the states with LFPN "correlated" and the RFPN "anti-correlated" with the MON; blue tones for the states with LFPN "anti-correlated" and the RFPN "correlated" with the MON. (B) depicts the decoded sequences of each correlation between an RSN and the MON. (DMN: default mode network; LFPN: left fronto-parietal network; RFPN: right fronto-parietal network; OPFN: occipito-parieto-frontal network; OPN: occipital posterior network; MON: medial occipital network; BG: basal ganglia).

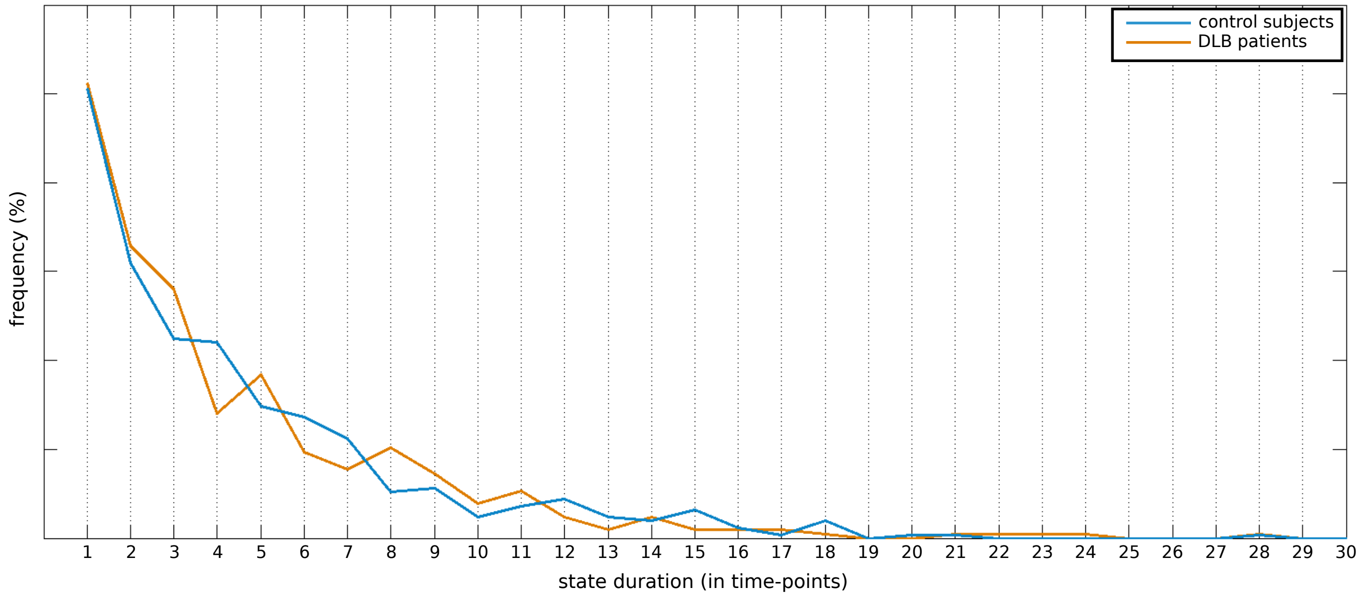

Figure S3. Distribution of the state duration in sequences $\hat{\mathbf{X}}_{\mathrm{MON}}$ for DLB patients and control subjects, in time points (TR = 3 seconds). (MON: Median Occipital Network).

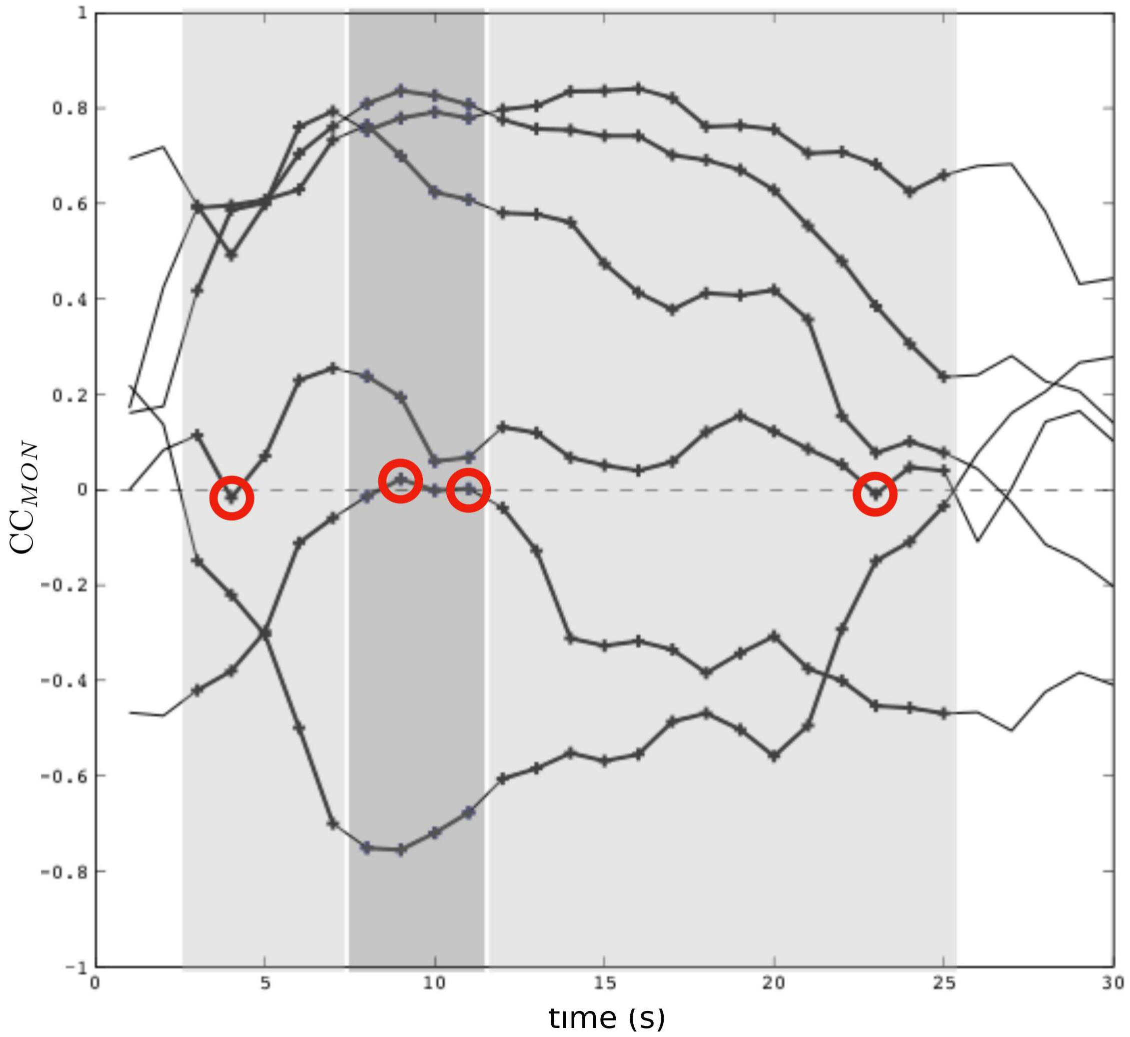

Figure S4. Distribution of the states for the 25 first seconds of the sequence, reported on the CC_{MON} for patient #1. This is a zoom on the Figure 4.(C).
